# Crab-Apple (*Mulus asiatica Nakai*) Peel Extract-Enhanced Potato Starch/κ-Carrageenan Bioactive Films: Structural Characterization, Antioxidant-Antimicrobial Efficacy, and Application in Meat Preservation

**DOI:** 10.3390/polym17101328

**Published:** 2025-05-13

**Authors:** Xiujie Lang, Ning Wang, Xuanzhe An, Cuntang Wang

**Affiliations:** 1 College of Food and Bioengineering, Qiqihar University, Qiqihar 161006, China; 2 Engineering Research Center of Plant Food Processing Technology, Ministry of Education, Qiqihar 161006, China

**Keywords:** potato starch, ethanol extract of crab-apple peel, complex film, antioxidant activity, antimicrobial activity, pork preservation

## Abstract

The development of biodegradable food packaging materials with active functionalities presents a sustainable alternative to conventional plastic films. This study developed a bioactive complex film through solvent casting technique using potato starch (PS) and κ-carrageenan (κC) as the matrix, incorporated with ethanol extract of crab-apple peel (EEC). Fourier-transform infrared analysis confirmed the formation of hydrogen bonds between the film-forming constituents. Scanning electron microscopy revealed that higher concentrations of EEC led to a relatively rough film surface. XRD indicated that the incorporation of EEC reduced the crystallinity of the potato starch. The addition of EEC significantly increased the a and b values of the complex film (*p* < 0.05), while the L value and opacity decreased significantly (*p* < 0.05). The TS, Young′s modulus, and WVP of the complex films decreased significantly with increasing EEC concentration (*p* < 0.05). The DPPH and ABTS radical scavenging abilities of PS-κC-EEC film were enhanced from 12.35% to 75.48% and from 10.26% to 72.52%, respectively. PS-κC-EEC film exhibited strong antimicrobial activity against *Staphylococcus aureus* and *Escherichia coli*. The application of the PS-κC-EEC film for the refrigerated preservation of pork demonstrated that the lipid oxidation level of wrapped pork was reduced. These results suggest that the fabricated PS-κC-EEC film could be utilized for the preservation of pork and extends its shelf life.

## 1. Introduction

Advanced food packaging systems serve as critical components in maintaining product integrity, extending shelf-life stability, and preserving nutritional profiles during distribution cycles [1]. Synthetic polymers have emerged as predominant materials in commercial food containment applications due to their cost-efficiency and versatile physicochemical properties [2]. Nevertheless, the petroleum-derived nature of these macromolecular compounds presents significant environmental challenges, with escalating volumes of post-consumer polymeric waste contributing to persistent ecological contamination through non-biodegradable accumulation [3]. This sustainability imperative has catalyzed intensive research into novel biopolymer-based alternatives exhibiting controlled biodegradation characteristics and reduced environmental persistence [4]. The application of natural polymer materials such as protein, starch and polysaccharides in food packaging is increasingly valued because of their renewability, abundance and degradability [2]. Starch is currently the most popular natural polymer food packaging material [5]. Compared with other natural polymer materials, starch is low-priced, non-toxic, renewable, degradable, and has good film-forming properties, so it can be used to develop natural biodegradable bio-based films as an important plastic substitute [6].

Among various starches, potato starch (PS) is considered ideal for developing into films because of its paste transparency, low pasting temperature, and high gelatin stability [7]. PS contains a relatively high content of amylopectin and phosphate groups. It has the advantages of a rapid rise in viscosity and easy expansion, which are favorable for food processing and utilization [8]. However, compared with plastics, a single PS film still has deficiencies in terms of water resistance and mechanical properties [9]. To conquer these deficiencies, the blending of starch with other biopolymers, such as carrageenan (CA), chitosan, glutin, xanthan gum, carboxymethyl cellulose, to form complexes has received extensive attention [10,11,12]. CA is a hydrophilic colloid extracted from algal species, and is widely utilized in the food industry as a water-retaining agent, emulsifier, and thickening agent [10]. Specifically, κ-carrageenan (κC) consists of potassium salts of sulfated polysaccharides composed of galactose and anhydrogalactose units [11]. Furthermore, κC exhibits exceptional gelation characteristics, high viscosity properties, and favorable film-forming capabilities. When forming complex films with starch, κC can significantly enhance the mechanical properties of the resulting complex films [12]. Wang et al. prepared complex films by blending κC with corn starch, and found that κC could enhance the film-forming capability of corn starch films [6]. To achieve the enhanced performance of food packaging materials, natural activators can be incorporated into the carrageenan/corn starch complex film to improve the antioxidant and antibacterial properties of the complex film [12]. Typically, plant polyphenolic substances (phenolic acids, tannic acids, anthocyanins, and flavonoids) are incorporated into starch complex films to enhance oxidative and antibacterial properties of food active packaging films, thereby preventing microbial infection and fat oxidation in food [12,13].

Plant polyphenols are secondary metabolites widely found in the roots, leaves and fruits of plants [11]. In recent years, a large number of research results have shown that polyphenols in plants have good antioxidant, antibacterial, antiviral, lipid regulation, and hypoglycemic effects [14]. Phenolic compounds in plants are usually extracted from waste products of fruit and vegetable processing [12]. Due to their powerful antioxidant and antimicrobial activities, they are often blended into starch-based complex films to develop active packaging to prolong the shelf life of foods [15,16,17,18].

Meat products, characterized by high lipid content, are prone to lipid oxidation during storage, leading to quality deterioration and reduced shelf life [19]. In recent years, active films incorporating plant-derived polyphenols have demonstrated growing potential in meat preservation [20,21]. Distillers′ dried grains with soluble protein–tea polyphenol complex films have been shown to prolong the shelf life of chilled pork by inhibiting lipid oxidation [22]. Similarly, watermelon rind pectin-based films loaded with kiwifruit peel extract significantly retarded lipid oxidation in chicken meat preservation [23]. Interestingly, the crab-apple is rich in bioactive substances such as polyphenols, flavonoids, and anthocyanins, and has antioxidant, anticancer, and other pharmacological effects; the polyphenols in crab-apple peel are also far higher than those in the pulp [24]. Although the nutritional value of crab-apple has been fully studied, there is no report so far on the application of antioxidant components in crab-apple peel in starch films. Therefore, PS was utilized as the base material, κC as the reinforcing agent, glycerol as the plasticizer, and crab-apple peel ethanol extract as the bioactive ingredient to fabricate an environmentally friendly and functional PS complex film. Additionally, the effects of crab-apple peel ethanol extract on the physicochemical properties, mechanical properties, microstructure, antioxidant properties and antibacterial activity of the complex film were evaluated, and the preservation effect of the complex film on pork was evaluated.

## 2. Materials and Methods

### 2.1. Materials

Crab-apple peel, pig lard, Qiqihar Tianyuan market. Photo starch, Heilongjiang Fufeng Starch Development Co., Ltd. (Harbin, China). κ-Carrageenan, (Food grade, Qingdao Dehui Marine Biotechnology Co., Ltd., Qingdao, China). *S. aureus* strains (ATCC29213), *Escherichia coli* (ATCC25922), College of Food and Biological Engineering, Qiqihar University (Qiqihar, China). DPPH (2,2-Diphenyl-1-picrylhydrazyl) and ABTS (2,2′-azino-bis- (3-ethylbenzothiazoline-6-sulfonic acid)), Sigma-Aldrich Chemical Co. (St. Louis, MO, USA). Other chemical reagents (analytical grade), Nanjin Chemical Reagents Co., Ltd. (Nanjin, China).

### 2.2. Extraction of EEC

The extraction method of crab-apple peel followed that of Gao et al. [16] with some modifications. The ethanol extract of crab-apple peel (EEC) was freeze-dried into a powder and stored at −20 °C for future use. The extraction rate of EEC was 9.24%. Detailed experimental methods are provided in the Supplementary Document.

### 2.3. Preparation of Films

The PS/кC-EEC films were prepared by the solution casting method according to the method of Riaz et al. [25]. The films were named PS/кC, 2%PS/кC-EEC, 4%PS/кC-EEC, and 6% PS/кC-EEC according to the amount of EEC added. Detailed experimental methods are provided in the Supplementary Document.

### 2.4. Characterization of the PS/к-C/EEC Films

#### 2.4.1. Mechanical Properties

The mechanical properties of films were determined by referring to the method of Huang et al. [26] with minor modifications. The thicknesses of the films were measured using a digital thickness micrometrer (SM-114, Teclock, Okaya, Japan). Detailed experimental methods are provided in the Supplementary Document.

#### 2.4.2. Water Vapor Permeability (WVP)

The determination of WVP was slightly modified with reference to Roy et al.′s method [27]. Detailed experimental methods are provided in the Supplementary Document.

#### 2.4.3. Color and Transparency

The color of the film sample was determined by use of a colorimeter (CR-10 Plus, Konica Minolta optics Co., Ltd., Shanghai, China) following the method of Gao et al. [16]. Detailed experimental methods are provided in the Supplementary Document.

The optical properties of the film samples were determined following the method of Sukhija et al. with minor modifications [28]. Detailed experimental methods are provided in the Supplementary Document.

#### 2.4.4. Structural Characterization of Films

The Fourier transform infrared (FT-IR) spectroscopy analysis (Spotlight 400, Perkin Elmer Co., Waltham, MA, USA) used the method of Wang et al. to determine the change of the wavelength between 4000 and 460 cm^−1^ [16]. Detailed experimental methods are provided in the Supplementary Document.

The method of Gao et al. [16] was used to analyze the thin films by X-ray diffraction (SmartLab, Rigaku Co., Tokyo, Japan). The XRD scan ranged from 5–80° (2θ), and the scan rate was 2°/min. Detailed experimental methods are provided in the Supplementary Document.

The complex films surfaces and cross sections of the complex films were observed by using a scanning electron microscope (SEM) (S-4300, Hitachi, Tokyo, Japan) [16]. Detailed experimental methods are provided in the Supplementary Document.

### 2.5. Total Phenolic Content and Antioxidant Activity of Films

Total phenolic content (TPC) quantification was performed via Folin–Ciocalteu spectrophotometry following established protocols [29], with the results standardized against gallic acid equivalents (GAE) and reported in mg GAE/g DW. Free radical scavenging capacity was evaluated through dual-spectrophotometric assays targeting DPPH and ABTS radical inhibition, as previously detailed in [29]. Detailed experimental methods are provided in the Supplementary Document.

### 2.6. Antimicrobial Activity

The antimicrobial activity of the film was determined by Gao et al. [16]. Detailed experimental methods are provided in the Supplementary Document.

### 2.7. Application of PC/кC-EEC Films in Pork Refrigeration and Preservation

The pork was cut into 5 g pieces. The sample groups were (1) control, (2) wrapped in control film, (3) wrapped in 2% PS/кC-EEC films, (4) wrapped in 4% PS/кC-EEC films, and (5) wrapped in 6% PS/кC-EEC films. Each film-wrapped pork sample was stored at 4 °C for 12 days and evaluated on day 0, day 3, day 6, day 9, and day 12. The peroxide value (PV) and 2-thiobarbituric acid reactive substance (TBARS) of pork were determined [28,30]. Detailed experimental methods are provided in the Supplementary Document.

### 2.8. Statistical Analysis

Each experiment was conducted in three independent replicates, and the data are presented as the mean ± standard deviation (SD). Duncan′s multiple range test, performed in SPSS 25 software (SPSS Inc., Chicago, IL, USA), was used to analyze the significance of the differences in the data. The experimental data were plotted with the help of Origin 2022 software (Microsoft, Redmond, Washington, DC, USA).

## 3. Results and Discussion

### 3.1. Physical Properties of the PC/κC-EEC Films

#### 3.1.1. Thickness and Mechanical Properties

**Figure 1:**
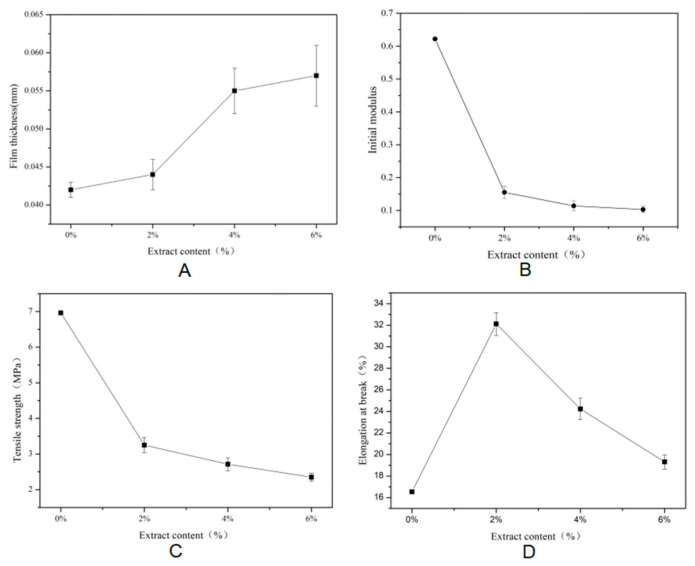
Thickness and mechanical properties of films with different concentrations of EEC. Note: (**A**) thickness; (**B**) Young′s modulus; (**C**), tensile strength; (**D**) elongation at break.

The mechanical properties of the film are characterized by thickness, Young′s modulus (YM), tensile strength (TS), and elongation at break (EB). The combination of EEC with к-carrageenan enhances the intermolecular forces of the film, resulting in a change in the mechanical properties of the film [31]. As shown in Figure 1A, the control film had the smallest thickness, the thickness of the film increased as the concentration of EEC increased, and the addition of EEC to the starch film produced a more complex matrix, which led to an increase in film thickness. This phenomenon may be attributed to the increased solid content concentration in the film-forming solution following the incorporation of EEC, which promoted the formation of thicker wet films during the solution-casting process, thereby resulting in a significant elevation in final film thickness post-drying [32]. Additionally, reactive functional groups in EEC, such as phenolic hydroxyl groups, established molecular interactions with starch molecular chains in the complex film. These interactions likely modified the intermolecular forces and spatial arrangement between starch chains, leading to an expanded interchain distance [32]. Consequently, the microstructural expansion of the film matrix occurred, which was macroscopically manifested as increased film thickness. Araújo et al. reported that an increase in the concentration of mango leaf extract led to a change in the conformation of the polymeric chains and an increase in the distribution of phytochemicals in the film, resulting in a thickening of the film [33]. A similar trend was reported by Qin et al., who found that the thickness of a cassava starch film increased with the increase in the content of anthocyanins from *Lycium ruthenicum Murr* [34].

YM is the basic measure of complex film stiffness. The higher the YM, the higher the stiffness of the film [13]. The YM was the largest for the control film, as shown in Figure 1B, and the YM of the complex film became lower and lower with the increase in EEC, which indicates that the addition of EEC could reduce the stiffness of the film and enhance the elasticity of the film. The TS and EB values of the films are shown in Figure 1C,D. The TS of the complex films was gradually weakened with the increase in the extract concentration, indicating that the addition of the extracts affects the densification of the film’s surface [17].

The incorporation of EEC significantly influences the mechanical properties of the complex film. With the increase in the addition of EEC, the EB of the complex film exhibited a trend of first rising and then declining, reaching its peak when the addition level was 2%. Although the EB gradually decreased when the level of EEC exceeded 2%, it was still higher than that of the control. This phenomenon could be attributed to the hydrogen bonding interaction between EEC and starch molecules. Moderate hydrogen bonding association enhances the flexibility of the film and increases the EB [32]. An excessive amount of EEC may lead to excessive intermolecular cross-linking, weakening the plasticizing effect. Meanwhile, the addition of EEC promotes the hardening of the original starch network structure, exhibiting the dual effects of plasticization and structural modification [32]. Relevant studies have provided evidence for the regulatory effect of such polyphenolic substances on the mechanical properties of starch-based complex films. When ethanol-extracted onion peel was added to the corn starch film, it reduced the TS and increased the EB [32]. Qin et al. [34] also revealed that the addition of *Lycium barbarum* anthocyanins to the PS film also leads to a decrease in TS.

#### 3.1.2. Analysis of Water Vapor Permeability (WVP)

**Figure 2:**
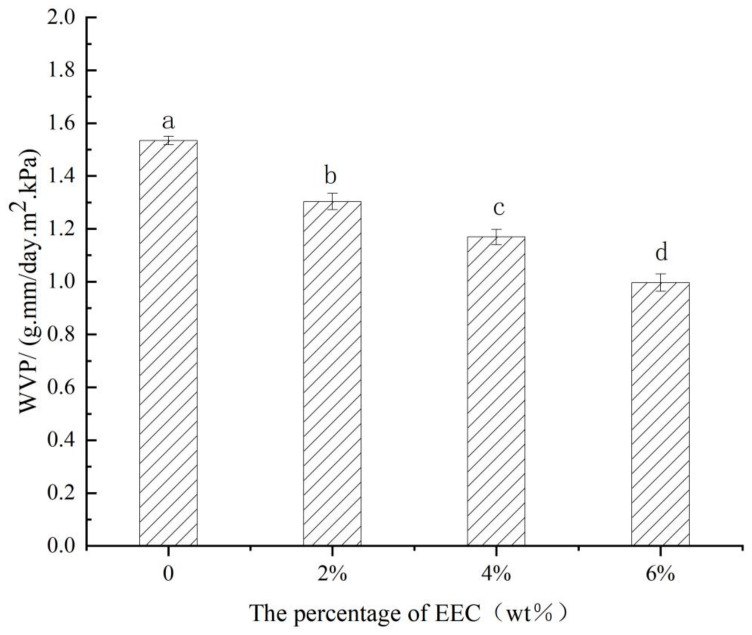
WVP of films with different concentration of EEC. Note: The same lower-case letters in the figure indicate significant differences (*p* > 0.05).

WVP serves as a critical indicator of the physical properties of food packaging films, reflecting their barrier efficacy against water vapor transmission. WVP is one of the factors that determine whether a film could prolong the shelf life of food [35]. Therefore, films with low WVP rate are more favored. WVP value is intrinsically governed by the structural and functional characteristics of the packaging material [25]. As shown in Figure 2, the control film had the highest WVP of 1.53 g mm/cm^2^ d KPa. With the addition of EEC, the WVP of the complex films exhibited a decline (*p* < 0.05). The decrease in the WVP of the PS/κ-C/EEC film can be attributed to the intermolecular interactions formed between starch and EEC, especially the hydrogen-bonding association effect [32]. In the complex film system, the molecular chains of starch and the polyphenolic structure of EEC form hydrogen bonds through hydroxyl groups and phenolic hydroxyl groups, which significantly enhances the intermolecular cross-linking degree, thereby inhibiting the penetration and diffusion of water vapor within the film [36]. Similarly, Qin et al. [34] revealed that incorporating *Lycium barbarum* anthocyanin inti potato starch films reduced the WVP. Tavares et al. [37] also found that interactions between starch and carboxymethylcellulose moieties limited the migration of the starch chains, thus reducing the permeability to water vapor.

#### 3.1.3. Color, Opacity, and Light Transmittance of the Film

The optical characteristics of packaging films significantly influence consumer perception and functional performance [35]. Chromatic analysis revealed concentration-dependent alterations in film appearance, transitioning from colorless transparency in control samples to yellowish hues upon EEC incorporation (Table 1). Progressive darkening was observed with increasing EEC concentrations, quantified by a significant decrease in lightness (L* value: 85.26 ± 0.54 to 78.17 ± 0.81, *p* < 0.05), likely attributed to reduced polymer-chain interactions and light scattering effects [38]. Concurrently, the opacity decreased proportionally to EEC loading, a phenomenon potentially associated with the polyphenolic chromophores (e.g., anthocyanins) present in the extract. These findings demonstrate statistically significant modifications in both chromatic parameters (a*, b*, L*) and light transmittance properties following EEC addition (*p* < 0.05), aligning with previous observations by Ren et al. [29], who reported analogous color intensification in anthocyanin-enriched к-Carrageenan/Carboxymethyl Cellulose films.

### 3.2. Scanning Electron Microscope (SEM)

**Figure 3:**
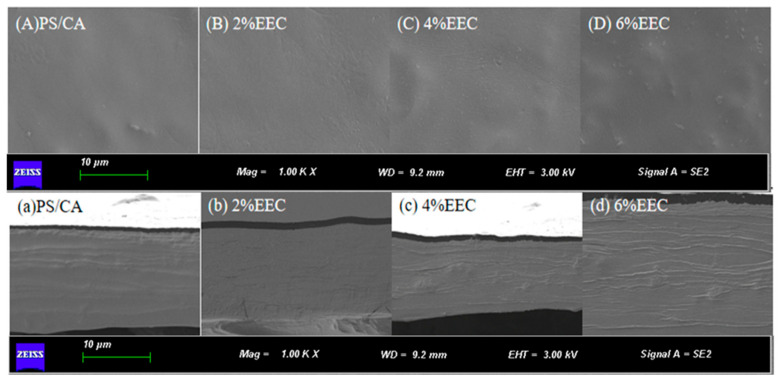
SEM micrographs of the surface and cross-section of films with different concentrations of EEC. Note: (**A**,**a**) surface and e cross-section of PS/кC film; (**B**,**b**) surface and f cross-section of 2% PS/кC/EEC film; (**C**,**c**) surface and g cross-section of 4% PS/кC/EEC film; (**D**,**d**) surface and h cross-section of 6% PS/кC/EEC film.

The structural configuration, morphological characteristics, and homogeneity of the complex film matrix critically govern the microstructural architecture of the complex films [29]. Figure 3 exhibits the SEM images of the surface and cross-section of the PS/κ-C and PC/κ-C/EEC complex films. The SEM images show that glycerol and carrageenan were well combined with PS, and the surface of the PS/кC film was smooth, organized, and structurally homogeneous, without bubbles or porosity. Figure 3B–D,b–d show that PS/к-C and EEC are tightly bound, thus forming a uniform surface on the surface and cross-section of the film. The binding effect of straight-chain starch to EEC was stronger than that of branched-chain starch, so PS/к-C with a high content of straight-chain starch binds well to EEC. Under the condition of low concentrations of EEC, there was no obvious surface aggregation in the films. When the EEC content was increased to 4% (Figure 3c), the cross-section of the film was much rougher than that of the other films, and some irregular shapes appeared, and some small particles appeared on the surface in Figure 3C. The gradual roughening of the surface may be attributed to the decrease in the solubility of the system when too much EEC was added, leading to the appearance of insoluble particles. Riaz et al. [25] also found that when Chinese chive root extract was added to chitosan film, the surface and cross-section of the film became rough. Pramitasari et al. [39] showed that when red dragon fruit peel anthocyanin extract was blended with tapioca starch, the film showed the appearance of insoluble granules.

### 3.3. Fourier Transform Infrared (FT-IR) Spectroscopy

**Figure 4:**
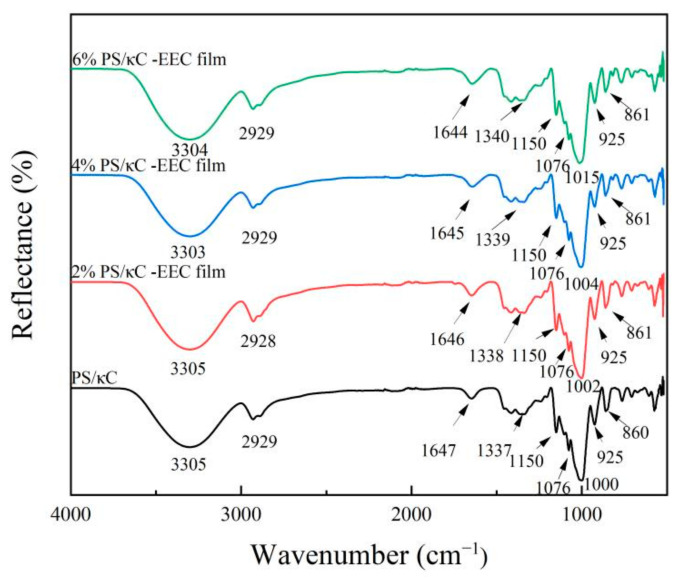
FT-IR spectra of PS/кC, 2% PS/кC/EEC, 4% PS/кC and 6% PS/кC/EEC films.

Infrared spectrograms show the interactions between molecules and functional groups within EEC films [30]. Figure 4 shows the infrared spectra of PS/к-C -EEC films; the FT-IR spectra reflect the interaction between EEC and PC molecules. In Figure 4, it is evident that the PS/к-C control films have significant absorption peaks at 3305 cm^−1^, 2929 cm^−1^, 1647 cm^−1^, 1000 cm^−1^, and 925 cm^−1^. The vibration at 3305 cm^−1^ was attributed to O–H stretching [16]. The O–H stretching seems to have a slight variation towards lower wave numbers, which indicates the presence of hydrogen bonding between the hydroxyl groups of the polymer components and the polyphenolic substances (EEC) [32]. The appearance of a small absorption peak at 2929 cm^−1^ is due to the C–H bending that was produced [15]. The absorption peak at 1647 cm^−1^ was due to asymmetric and symmetric expansion vibrations at C=O [32]. The results show that the characteristic peak was related to the crystallinity of the starch, and the decrease in crystallinity led to an increase in the intensity of the characteristic peak [40]. The small absorption peak at 1337 cm^−1^ is due to the band vibrations of the C–H and CH_2_ deformation [40].

The bands from 1000^−1^ to 1150 cm^−1^ correspond to the characteristic pyranose ring of glucose residues [35]. The absorption peak at 925 cm^−1^ is generated by the out-of-plane bending vibrations of the olefins [32]. Upon the addition of EEC to the PS/κ-C complex, the hydroxyl (-OH) and other characteristic peaks in the FTIR spectrum shifted to right wavenumbers, suggesting that EEC modified the hydrogen bonding interactions within the PS/κ-C matrix. The research by Qin et al. [34] yielded similar conclusions. After introducing anthocyanins from *Lycium ruthenicum* into the cassava starch system, the two formed a supramolecular complex through intermolecular hydrogen bonding, significantly optimizing the physicochemical properties of cassava starch.

### 3.4. X-Ray Diffraction (XRD)

**Figure 5:**
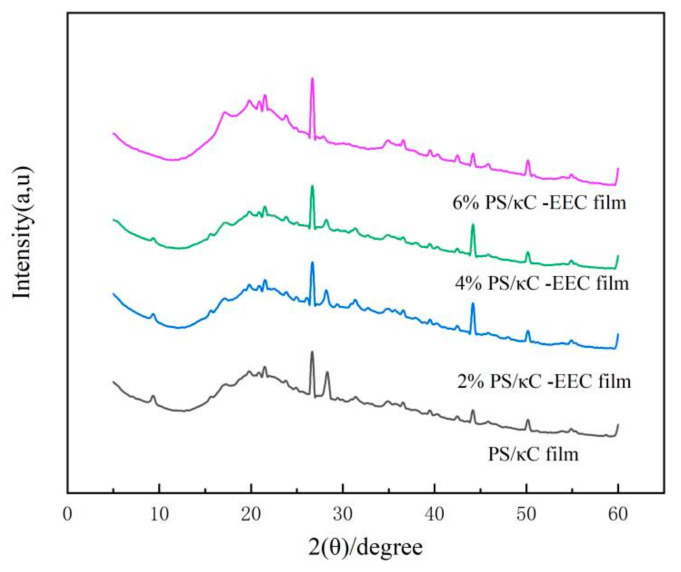
XRD patterns of PS/кC, 2% PS/кC/EEC, 4% PS/кC/EEC and 6% PS/кC/EEC films.

Thermoplastic starch films are semi-crystalline, and their crystal structure can be recognized by X-ray diffraction patterns [11]. Figure 5 shows the XRD patterns and corresponding crystallinity of PS/кC films containing different addition levels of EEC. The PS/кC films show absorption peaks at 9.32°, 20.84°, 21.55°, 26.84°, 28.38°, 44.27°, and 50.28°. During storage, the potato starch within the complex film underwent a retrogradation process, manifesting a broad diffraction peak in the vicinity of 20°. Specifically, the diffraction peaks observed at 20.84° and 21.55° are associated with the recrystallization phenomenon, indicating that the PS/κC film exists in a recrystallized state.

As the concentration of EEC increased, the diffraction peak at 9.3° gradually disappeared, revealing that the addition of EEC modified the structure of the complex film, formed new hydrogen bonds, and reduced the crystallinity of the film [41]. The highest diffraction peak at 26.84° was observed in PS/кC films and EEC-enriched films, which was attributed to the presence of potassium ions in the κ-carage, resulting in the characteristic peak of potassium ion at 2θ = 26.84° [11]. The diffraction peak of PS/кC film at 2θ = 28.38° gradually disappeared with the addition of the EEC diffraction peak, which may be related to the interference of EEC in the diffraction of the KCL crystal [11]. A similar conclusion was reached by Bao et al. [42], who found that mixing blueberry anthocyanins and chondroitin sulfate in potato starch resulted in hydrogen bonding with potato starch. These results indicate that the addition of EEC can significantly enhance the intermolecular interactions within the complex membrane, including secondary bond forces such as hydrogen bonds and van der Waals forces [17]. This strengthened intermolecular force not only endows the complex films with excellent biocompatibility, but also drives the evolution of the film′s microstructure towards orderliness and densification [29]. This conclusion is consistent with the results of FT-IR analysis, which reveals changes in the vibration characteristics of functional groups and an enhancement of intermolecular interactions [11].

### 3.5. Antioxidant Activity of the PS/κC-EEC Films

**Figure 6:**
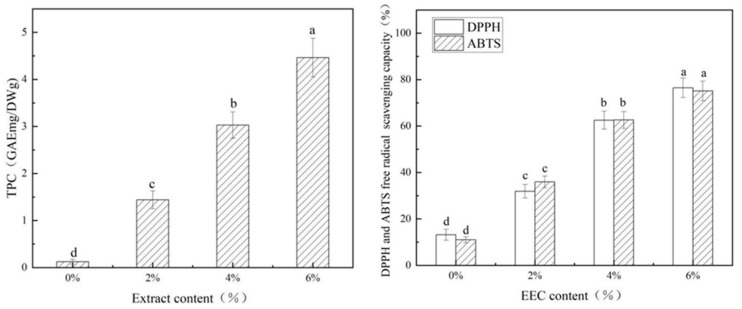
Total phenolic content and free-radical scavenging activity (DPPH and ABTS) of the PS/кC, 2% PS/кC/EEC, 4% PS/кC/EEC and 6% PS/кC/EEC films. Note: The same lower-case letters in the figure indicate significant differences (*p* > 0.05).

During the storage and circulation of food, the oxidation reaction is one of the key factors leading to the deterioration of its quality [2]. The oxidation reaction not only causes color changes in food, affecting consumers′ purchasing desire, but also damages the nutritional components of food, resulting in a reduction in nutritional value [3]. Total phenolic content (TPC) is one of the most important indicators of antioxidant performance; usually, the higher TPC, the better the antioxidant activity [11]. The TPC of the PS/кC/EEC film is shown in Figure 6. The TPC of the PS/κC film was the lowest. With the gradual addition of EEC, the TPC of the film showed an increasing trend, and a significant dose–response relationship was presented, that is, the higher the addition level of EEC, the greater the increase in the TPC of the complex film. Gasti et al. [43] also found that *Phyllanthus reticulatus* also increased the TPC of the films. Wang et al. [11] derived a significantly increased TPC in films by incorporating grape seed ethanol extract into corn starch.

Free radicals could lead to spoilage and a loss of nutritional quality in packaged foods. Therefore, antioxidant capacity is very important for food packaging film [44]. DPPH and ABTS radical scavenging were widely used as a common method to test the antioxidant activity of complex films [11]. The DPPH and ABTS radical scavenging data of PS/кC/EEC are shown in Figure 6. The DPPH and ABTS radical scavenging values of the PS/кC films were lower, at 13.2% and 11%. The DPPH and ABTS radical scavenging rates of the complex films increased when EEC was added and increased with the concentration of EEC, which was attributed to the efficacy of the natural extract EEC. Many studies have also confirmed that natural extracts can effectively scavenge free radicals [11,17]. Similarly, Kumar et al. [45] found that pineapple peel extract was effective in enhancing the free radical scavenging rate of corn starch films. Our results suggest that the incorporation of EEC into PS/кC films has great potential to yield an antioxidant package that can retard lipid oxidation and prolong the shelf life of foods.

### 3.6. Antibacterial Properties of PS/кC/EEC Films

Antimicrobial properties are important properties of food packaging films, helping to reduce microbial contamination in food. Bacteriostatic performance is evaluated by measuring the diameter of the bacteriostatic zone [11]. Usually, the bacteriostatic performance of the film sample is evaluated by measuring the inhibitory effect against foodborne bacteria [17]. This study selected *Staphylococcus aureus* (Gram-positive bacterium) and *Escherichia coli* (Gram-negative bacterium), two prevalent foodborne bacterial strains, as indicator organisms to evaluate the antibacterial efficacy of PS/κC/EEC films against distinct bacterial types. The antibacterial properties of the PS/кC/EEC film is shown in Table 2.

The complex films incorporated with EEC exhibited significantly higher antibacterial activity against *S. aureus* compared to *E. coli*. The antibacterial values of PS/кC film against *E. coli* and *S. aureus* are 6.86 ± 0.02 mm and 7.02 ± 0.04 mm. The addition of EEC can significantly increase the diameter of the antibacterial zone of the complex film, and there is a concentration-dependent relationship between them. It is worth noting that there was no significant difference (*p* > 0.05) in the diameters of the antibacterial zones of the 4% and 6% PS/κC/EEC complex films. This may be due to the synergistic antibacterial effect of κC and EEC; κC forms a physical barrier, and the polyphenolic substances in EEC disrupt the cell membranes of bacteria and interfere with their metabolism, jointly inhibiting the growth of foodborne bacteria. When the concentration of EEC reaches 4%, the antibacterial activity approaches saturation, so increasing it to 6% does not significantly enhance the effect. EEC was rich in polyphenolic substances, which can interfere with the growth and reproduction of microorganisms by increasing the permeability of their cell membranes and inhibiting their energy metabolism, thereby enhancing the antibacterial property of the PS/κC complex film [11,17]. Similarly, adding rosemary extract to cassava starch film also yielded similar antimicrobial properties [46]. Riaz et al. [25] found that leek root extract can also enhance the antibacterial properties of the film.

### 3.7. Application of PS/κC/EEC Film in Cold Storage and Fresh-Keeping of Pork

**Figure 7:**
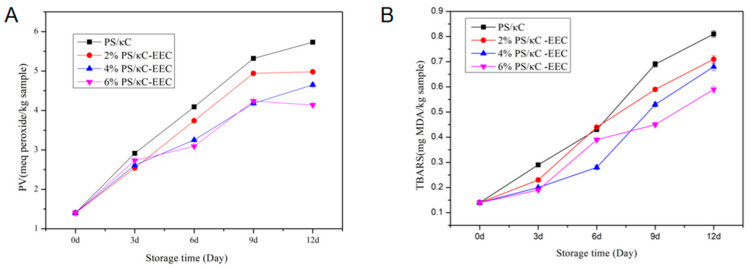
Change in PV (**A**) and TBARS (**B**) values of pork during storage at 4 °C.

PV reflects the total peroxide content and the degree of peroxide in the sample [17,30]. According to Figure 7A, the PV value of pork rose with the extension of storage time, indicating the lipid oxidation of the sample during storage. On day 0, the PV of the pork was 1.4 meq peroxides/kg. The PV value of the pork wrapped with the PS/κC/EEC film was significantly higher than that of the pork wrapped with the PS/κC film. The higher the concentration of EEC in the complex film, the lower the PV value of the pork wrapped in the complex film. In contrast, the 6%PS/кC/EEC film has strong antioxidant properties. The PV of the pork wrapped in the film was 1.4 meq peroxides/kg on day 0, and the PV of the samples wrapped in the 2% PS/к-C/EEC film and the 6% PS/кC/EEC film on day 9 was higher than that on day 12, which may be due to the rapid decomposition of the unstable peroxides produced by the oxidation of the pork into secondary products [47]. The results explain that the addition of EEC into the PS/кC film delayed the lipid oxidation of pork. Similarly, Han et al. [23] showed that the active film infiltrated with kiwi peel extract could reduce the PV value of t=wrapped chicken and delay lipid oxidation in chicken thighs.

TBARS measures lipid secondary oxidation products, mainly hydrocarbons, which are the main cause of meat flavor deterioration and odor production [48]. With the extension of storage time, the value of TARS in pork increased continuously, indicating that lipid oxidation occurred during storage (Figure 7B). On day 0, the TBARS value of pork in the control was the lowest, with an initial value of 0.14 mg MDA/kg. The TBARS value of pork wrapped in PS/кC/EEC complex film was significantly lower than that of the control. The higher the EEC concentration in the complex film, the slower the increase in TBARS in the wrapped pork. On days 6, 9 and 12, TBARS in pork wrapped with 6% PC/кC/EEC film were lower than those in pork wrapped with 0% PS/кC/EEC film. Therefore, due to the antioxidants present in the EEC, the metals of lipoxygenase can be chelated, inhibiting lipid oxidation delay. In contrast, the 6%PS/кC/EEC film has strong antioxidant properties. It is worth noting that the TBARS values of all the samples increased during storage. Portugal Zegarra et al. [49] found that chitosan films containing acerola residue extract were applied to chicken thighs. These results suggest that the PS/кC/EEC complex film could restrain lipid oxidation in pork.

## 4. Conclusions

This study successfully demonstrates the fabrication and functional efficacy of a bioactive complex film based on PS and κC incorporated with crab-apple peel ethanol extract (EEC) for active food packaging applications. Structural characterization revealed that the hydrogen bonding interactions between EEC and the polysaccharide matrix altered the crystallinity and morphology of the films. A high concentration of EEC leads to a decrease in the crystallinity of starch and an increase in the surface roughness of complex films. Furthermore, the incorporation of EEC enhances the mechanical properties and WVP of the complex film to some extent, and leads to significantly enhanced bioactivity. Notably, the PS-κC-EEC film displayed a potent dual-action performance, achieving 75.48% DPPH and 72.52% ABTS radical scavenging capacities, while demonstrating marked antimicrobial efficacy against foodborne pathogens. Practical validation through refrigerated pork preservation trials confirmed the material’s effectiveness. These findings substantiate that EEC incorporation effectively helps to develop PS-kC complexes into active packaging materials, addressing both meat preservation challenges. Future research should focus on optimizing mechanical–thermal stability through crosslinking strategies and evaluating scalability for commercial meat packaging applications.

## Figures and Tables

**Table 1:** Color, optical properties, and opacity of PS/кC/EEC films with different concentrations of EEC.

Extract Concentration	L*	*a* *	*b* *	Opacity/%	Picture
0%	85.26 ± 0.54 ^a^	−0.10 ± 0.01 ^d^	1.29 ± 0.09 ^d^	5.57 ± 0.02 ^a^	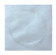
2%	84.97 ± 0.16 ^b^	3.36 ± 0.09 ^c^	7.18 ± 0.09 ^c^	4.14 ± 0.02 ^d^	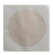
4%	80.36 ± 0.21 ^c^	7.17 ± 0.26 ^b^	9.57 ± 0.12 ^b^	4.38 ± 0.08 ^c^	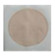
6%	78.17 ± 0.81 ^d^	9.12 ± 0.52 ^a^	11.22 ± 0.43 ^a^	4.85 ± 0.03 ^b^	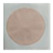

Values are given as mean ± standard deviation. Different lowercase letters in the same column indicate significant differences (*p* < 0.05).

**Table 2:** Antibacterial properties of PS/кC/EEC films with different concentrations of EEC.

Extract Concentration	Diameter of the Bacteriostatic Circle (mm)	
	*E*. *coli*	*S*. *aureus*
0%	6.86 ± 0.02 ^Bc^	7.02 ± 0.04 ^Ac^
2%	8.47 ± 0.25 ^Bb^	8.99 ± 0.28 ^Ab^
4%	10.92 ± 0.31 ^Ba^	11.92 ± 0.38 ^Aa^
6%	11.20 ± 0.52 ^Ba^	12.05 ± 0.42 ^Aa^

Values are given as mean ± standard deviation. Different lower-case letters in the same column indicate significantly differences (*p* < 0.05). Different capital letters in the same row indicate significant differences (*p* < 0.05).

## Data Availability

The original data used in this research are included in the article.

## Supplementary Materials

The following supporting information can be downloaded at: https://www.mdpi.com/article/10.3390/polym17101328/s1. We put the detailed experimental methods in the Supplementary File.

## Abbreviations

Potato starch (PS); κ-carrageenan (κC); ethanol extract of crab-apple peel (EEC); tensile strength (TS); elongation at break (EB); water vapor permeability (WVP); scanning electron microscopy (SEM); total phenolic content (TPC).
