# Supplementary material for: Crab-Apple (Mulus asiatica Nakai) Peel Extract-Enhanced Potato Starch/κ-Carrageenan Bioactive Films: Structural Characterization, Antioxidant-Antimicrobial Efficacy, and Application in Meat Preservation
Source: Polymers (Basel). 2025 May 13;17(10):1328. doi: 10.3390/polym17101328 (PMC12115219; doi:10.3390/polym17101328)
Supplement: Supplementary file 1 [file polymers-17-01328-s001.zip › polymers-3613233-supplementary.pdf]

Supplementary File

S1. Materials and methods

S1..1. Materials

Crab-apple peel, pig lard, Qiqihar Tianyuan market. Photo starch, Heilongjiang Fufeng Starch Development Co., Ltd (Harbin, China). κ-Carrageenan, (Food grade, Qingdao Dehui Marine Biotechnology Co. , Ltd). S. aureus strains (ATCC29213), Escherichia coli (ATCC25922), College of Food and Biological Engineering, Qiqihar University (Qiqihar, China). DPPH(2,2-Diphenyl-1-picrylhydrazyl) and ABTS (2,2′-azino-bis-(3-ethylbenzothiazoline-6-sulfonic acid)), Sigma-Aldrich Chemical Co. (St. Louis, MO, USA). Other chemical reagents (analytical grade), Nanjin Chemical Reagents Co., LTD.

S1.2. Extraction of EEC

The extraction method of crab-apple peel followed that of Gao et al. [16] with some modifications. The crabapple peel was crushed using a multi-functional crusher (GX-220, Zhejiang Gaoxin Industry and Trade Co., LTD., Yongkang, China). After crushing, 70% ethanol was added to the crab-apple peel. The extract was obtained after 2 hours of extraction at room temperature. The extract was centrifuged at 6000 r/min. The residue was extracted three times using the same method. Then, the supernatants from the three extractions were mixed and transferred to a rotary evaporator (2L-ARE, Shanghai Haozhuang Instrument Co., Shanghai, China). The supernatant was concentrated at 50 ℃. The concentrated solution was lyophilized in a vacuum freeze-dryer (2.5 L freeze dryer, Labconco Company, Kansas City, MO, USA). The resulting powder was the ethyl acetate extract (EEC) of the crab-apple peel. The EEC was stored refrigerated at 4 ℃. The extraction yield of the ethanol extract of the crab-apple peel was 9.24%.

S1.3 Preparation of Z/CA/GSEE Composite Film

PS/κC -EEC films were prepared by solution-casting according to the method of Riaz et al. with slight modifications [19]. PS gelatinized solution was prepared by dissolving 4 g PS in 200 g distilled water and gelatinizing it for 30 min at 90 ℃. Then, 1.8 g κ-carrageenan was added into the PS gelatinized solution with stirring for 30 min at 90 ℃. After that, different weights of EEC (0, 1, 3, and 5 wt. % on PS basis) were added into PS/κC film-forming solutions with stirring for 30 min at 90 ℃. Then, 1.8 g glycerol was added as a plasticizer to the PS/κC -EEC film-forming solution and stirred for 30 min at 90 ℃, using hot water to complement the evaporated water. The PS/κC -EEC film-forming solution was sonicated at 90 ℃ for 30 min to remove bubbles. A 120 mm-diameter polyethylene ring was fixed on a glass plate covered with release paper. The PS/κC -EEC film-forming solutions (25g) were poured onto the polyethylene ring and fixed for 15 min. The glass plates with film-forming solutions were placed in a blast drying oven and dried at 40 ℃ for 12 hours. Then, the films were carefully removed from the glass plates. The films were placed in a dryer at 25 ℃ with a relative humidity of 56.8% (NaBr saturated solution) and were balanced for 72 h to determine the parameters of the films. The films were placed in a desiccator with a relative humidity of 57.57% (NaBr saturated solution) at 25 ℃ for 72 h, and then the indexes of the film were determined. Finally, the prepared composite films containing 0, 2, 4, and 6 wt. % EEC were designated PS/κC, 2%PS/κC -EEC, 4%PS/κC -EEC, and 6%PS/κC -EEC films, respectively.

S1.4. Characterization of the PS/κ-C/EEC films

S1.4.1. Mechanical properties

The mechanical properties of films were determined by referring to the method of Huang et al. with minor modifications [20]. The tensile strength (TS), Young's modulus (YM) and elongation at break (EB) of the films were measured by a texture analyzer (TA. XT plus, Stable Micro System company,

Surrey, UK). All films were cut into strips of 6 cm×2 cm, and the test speed and initial clip spacing were 2 mm/s and 20 mm, respectively. The thicknesses of the films were measured using a digital thickness micrometrer (SM-114, Teclock, Okaya, Japan).

S1.4.2. Water vapor permeability (WVP)

The determination of WVP was slightly modified with reference to Roy et al.'s method [27]. We put 10 g of anhydrous CaCl2 into a bellows, dried it at 110 ℃ for 2 h, and put it into a weighing bottle (35 mm×90 mm). The mouth of the weighing bottle was covered with the prepared film sample (35 mm×90 mm), and the weighing bottle was re-discharged with oil-dried anhydrous CaCl2, which was sealed and put into the dryer with distilled water at the bottom. The mass of the weighing bottle was measured every 24 h for 10 consecutive days. The WVP of the film was calculated according to Equation (1),

$$WVP = \frac{W}{t \times A} \times \frac{X}{\Delta P} \qquad (1)$$

In this formula the following pertains: W, the total mass of the bottle after film sealing, (g); t, the testing time (s); A, the permeable film area (m2); X, the film thickness (m); ΔP, vapor pressure difference between two sides of the film (1583 Pa at 25 ℃).

S1.4.3. Color and transparency

The color of the film sample was determined by a colorimeter (CR-10 Plus, Konica Minolta optics Co., Ltd, Shanghai, China) following the method of Gao et al. [18]. Before measurement, a standard plate was used for calibration (L0= 101.25, a0=-2.20, b0= 6.68). Each sample was measured three times, and the results were averaged.

The optical properties of the films samples were determined following the method of Sukhija et al. [21] with minor modifications. The film sample was cut into a rectangle of 10 mm×30 mm and placed close to the inner wall of the empty cuvette. The empty cuvette was used as a reference to determine the absorbance value of the sample at 600 nm. The opacity is calculated according to Formula (2); each sample was measured three times, and the results were averaged.

$$\text{Opacity}=\frac{A_{600}}{X} \qquad (2)$$

Here, A600 is the absorbance value at wavelength 600 nm; X is the film thickness, mm.

Appearance: The composite film was placed on A4 white paper and the image of the composite film was taken.

S1.4.4. Structural characterization of films

The Fourier transform infrared (FT-IR) spectroscopy analysis (Spotlight 400, Perkin Elmer Co., Waltham, MA, USA) was performed via the method of Wang et al. to determine the change in the wavelength of the film between 4000cm-1 and 460cm-1[16].

The method of Gao et al. [16] was used to analyze the thin films via X-ray diffraction (SmartLab, Rigaku Co., Japan). The XRD scan ranged from 5 to 80°(2θ), and the scan rate was 2°/min.

The composite film surface and a cross-section of the composite film were observed using a scanning electron microscope (SEM) (S-4300, Hitachi, Japan) [16].

S1.5. Total phenolic content and antioxidant activity of films

Total phenolic content (TPC) quantification was performed via Folin–Ciocalteu spectrophotometry following established protocols [29], with the results standardized against acid equivalents (GAE) and reported as mg GAE/g DW. Free radical scavenging capacity was evaluated through dual-spectrophotometric assays targeting DPPH and ABTS radical inhibition, as previously detailed in [29].

S1.6. Antimicrobial activity

The antimicrobial activity of the film was determined by Gao et al. [16]. The disk diffusion test was used to detect the antibacterial activity of the PS/κC-EEC film against Staphylococcus aureus and Escherichia coli. The film was made into a sterile filter paper with a diameter of 10 mm, immersed in the film liquid, cut off, and placed on a medium containing 0.1 mL of bacteria. The petri dishes were inverted in an incubator at a temperature of 37 ℃ for 24 hours. The diameter of the inhibition circle was measured after completion.

S1.7. Application of PS/κC -EEC films in pork refrigeration and preservation

The pork was cut into 5 g pieces. The sample groups were (1) control, (2) wrapped in control film, (3) wrapped in 2% PS/κC-EEC films, (4) wrapped in 4% PS/κC-EEC films, and (5) wrapped in 6% PC/κC-EEC films. Each film-wrapped pork sample was stored at 4 ℃ for 12 days and evaluated on day 0, day 3, day 6, day 9, and day 12. The peroxide value (PV) and 2-thiobarbituric acid reactive substance (TBARS) of pork were determined [28, 30]. Each sample (2g) was added to 30 mL acetic acid/chloroform (3:2, v/v) solution and mixed at 300 RPM for 30 min. After adding 30 mL of distilled water, 1 mL of 1% starch was added and the solution was titrated with 0.01 N of sodium thiosulfate solution. PV is measured in milliequivalents (meq) of hydrogen peroxide per kilogram of sample.

Here, 2 g of pork and 10 mL of 7.5% trichloroacetic acid were shaken together and the mixture was homogenized for 3 min. After filtration, the mixture was placed in a water bath at 95 ◦C for 45 min and centrifuged (3500 × g, 4 ◦C) after the addition of 0.02 M 2-thiobarbituric acid solution (1:1, v/v). Absorbance was measured at 539 nm. The TBARS value was expressed as mg malondialdehyde (MDA)/kg sample.

S1.8. Statistical Analysis

Each experiment was conducted in three independent replicates, and the data are presented as the mean ± standard deviation (SD). Duncan's multiple range test was performed SPSS 25 software (SPSS Inc., Chicago, IL, USA) to analyze the significance of the differences in the data. The experimental data were plotted with the help of Origin 2022 software (Microsoft, WSU, USA).
